# Medical physics in the Nordic countries: collaboration and future challenges – report from the Nordic Association for Clinical Physics 2026 Symposium

**DOI:** 10.2340/1651-226X.2026.46249

**Published:** 2026-07-30

**Authors:** Emelie Adolfsson, Jan Seppälä

**Affiliations:** a Department of Health, Medicine and Caring Sciences, Linköping University, Linköping, Sweden; b Clinical Department of Medical Radiation Physics, Region Östergötland, Linköping, Sweden; c Department of Radiotherapy, Kuopio University Hospital, Kuopio, Finland

The Nordic Association for Clinical Physics (NACP) held its triennial symposium in Gothenburg, Sweden, from 24 to 26 March 2026. Under the theme ‘Collaboration and future challenges’, the symposium brought together medical physicists from radio-therapy, radiology, nuclear medicine, and magnetic resonance, providing a comprehensive overview of emerging technologies and their translation into clinical practice in the Nordic region. The symposium comprised plenary sessions, parallel scientific sessions, workshops, and poster presentations, offering a platform for both emerging research and clinical implementation. A recurring message throughout the symposium was the role of the medical physicist not only as a physics expert but also as a key contributor to safe and effective patient care. The meeting captured both scientific advances and practical challenges facing medical physicists today.

The symposium opened with an engaging retrospective on the history of NACP, founded in 1962, presented by the current president, Toni Ihalainen (FI). He highlighted the association’s early international impact, including contributions to developments that later formed international standards such as the International Atomic Energy Agency (IAEA) dosimetry protocol. Following a 12-year period of inactivity, the association was revitalized with the approval of a new constitution by the boards of the Nordic national member organizations in 2004. For physicists whose professional experience is largely rooted in the 21st century, this historical perspective provided a valuable and, in many ways, eye-opening insight into the legacy and evolution of the field.

A recurring theme of the two most recent NACP symposia has been the introduction of artificial intelligence (AI) in medical physics, and the discussions at the 2026 symposium reflected a clear maturation of the field. Whereas earlier meetings emphasized methodological development, the 2026 symposium shifted the focus toward clinical implementation and practical deployment. This transition was exemplified in plenary lectures by Mika Kortesniemi (FI), ‘The role of the Medical Physicist with respect to AI’, and Mark Gooding, ‘RoboRadOnc: The future of radiation therapy?’. Together, these presentations underscored both the evolving role of the medical physicist and the broader trajectory of the field, pointing toward increasingly data-driven, clinically grounded, and automated approaches to patient care.

## Scientific themes emerging from NACP 2026

Scientific contributions from proffered papers and posters reflected a broad range of topics, including predictive modeling of treatment-related toxicity, ultra-high dose rate radiotherapy, and target motion management.

Reirradiation is becoming an increasingly relevant component of radiotherapy, driven by several converging developments. Improved cancer survival has led to a growing population of patients presenting with local recurrences or new primary tumors in previously irradiated regions. At the same time, advances in treatment delivery, imaging, and dose planning have enabled more precise approaches, allowing reirradiation to be considered in situations where it was previously deemed too high risk. In this context, the plenary contribution by Ane Appelt was particularly timely. As a key figure in shaping international standards and research agendas in reirradiation, her presentation provided a comprehensive overview of current clinical practice alongside ongoing research efforts, highlighting both the opportunities and the remaining challenges in this evolving area [1].

A notable development is the growing emphasis on standardization and multi-institutional collaboration. While data-driven approaches have been discussed in earlier meetings [2], the 2026 symposium placed greater focus on structured clinical data, national registries, and coordinated efforts to support consistent practice. The KaSARi (Key advances in Standardizing, Automating, and predicting Risks in radiotherapy) initiative in Finland represents a particularly ambitious example of this development. Unlike a conventional retrospective registry, KaSARi is designed as a prospective national infrastructure integrated into routine clinical workflows, linking DICOM-RT data with toxicity and patient-reported outcomes [3]. The framework aims to provide a sustainable foundation for nationwide outcome evaluation, harmonization of radiotherapy practices, and future development of predictive and AI-based methods. In Denmark, a national collaboration has focused on patient safety through systematic analysis of adverse events, resulting in an updated framework for classification and risk assessment of unintended incidents in radiotherapy [4]. Beyond improving consistency in reporting, the initiative provides a mechanism for shared learning across institutions and illustrates how quality improvement increasingly relies on structured national collaboration rather than local experience alone. These efforts illustrate how future progress in medical physics will depend on robust infrastructures for collecting, standardizing, and actively using clinical data in practice.

As radiotherapy becomes increasingly advanced and data driven, greater attention must be paid not only to technical capabilities but also to their limitations, uncertainties, and clinical consequences. Several contributions highlighted the increasing complexity of modern radiotherapy and the need to evaluate both performance and robustness. Advances in MRI-based segmentation demonstrated that future clinical adoption of AI depends not only on geometric accuracy but also on the ability to identify when predictions are uncertain [5]. The study showed that improved uncertainty assessment and calibration can increase confidence in automated contouring by highlighting regions where manual review may be warranted, thereby supporting safer integration of AI into clinical radiotherapy workflows. A similar focus on uncertainty extends to other parts of the treatment chain. Differences between dose calculation algorithms become increasingly relevant as treatment plans grow more complex, underscoring the importance of understanding algorithm-specific limitations when evaluating highly modulated techniques [6]. In parallel, a comparison of volumetric modulated arc therapy and three-dimensional conformal radiotherapy for whole-breast treatment including simultaneous boost demonstrated improved dose conformity with VMAT but also illustrated trade-offs in organ-at-risk doses, emphasizing that more advanced techniques do not automatically translate into overall clinical benefit and must be evaluated in terms of clinical balance [7].

The interplay between complexity and reliability was also evident in quality assurance and dosimetry studies. An evaluation of starshot methods for isocenter size determination showed that EPID-based approaches can achieve comparable accuracy to traditional film-based approaches. Importantly, the work also demonstrated that the choice of beam collimation influenced the estimated isocenter size, highlighting how quality assurance results may depend not only on machine performance but also on the methodology used for assessment [8]. In ophthalmic brachytherapy using ^125^I seeds, accurate dose verification remains challenging because of the steep dose gradients and short source-to-detector distances involved [9]. These findings serve as a reminder that improvements in treatment precision must be matched by equally rigorous approaches to dosimetry and quality assurance. In highly localized treatments such as ophthalmic brachytherapy, even small measurement uncertainties may have a substantial impact on dose verification. Efforts toward more individualized treatment approaches were also reflected in radionuclide therapy, where pre-treatment prostate-specific membrane antigen positron emission tomography imaging was explored as a means of estimating absorbed doses during subsequent ^177^Lu PSMA therapy [10]. While substantial variability remained, particularly for tumor lesions, the results illustrate ongoing efforts to move toward patient-specific treatment planning in molecular radiotherapy.

Proton therapy has featured prominently at previous NACP meetings [11–13], yet in 2026 only two contributions were presented: clinical implementation of a dual-energy CT workflow [14] and the Norwegian Proton and Radiotherapy Registry [15]. With proton therapy centers now operational in Sweden, Denmark, and Norway, one might have expected a wider range of collaborative projects and data-sharing initiatives to be represented. The relatively limited representation of proton therapy may indicate that many ongoing collaborative activities are now being communicated through dedicated proton therapy networks, reflecting the maturation of the field within the Nordic region.

## Summary

In summary, the radiotherapy contributions at NACP 2026 reflect a field in transition. The key challenges are shifting from the development of technologically advanced solutions to their implementation, evaluation, and refinement in clinical practice. This includes systematic learning from previous treatments, structured follow-up of patient outcomes, and the growing use of clinical data to guide decision-making. The increasing integration of data-driven approaches and AI-based models further supports the move toward more individualized and adaptive treatment strategies. In this context, the role of medical physicists is evolving toward a broader clinical and integrative role. This development places new demands on the profession, not only in clinical practice but also in education and training, which must adapt to prepare future physicists for these expanded responsibilities. At the same time, it remains essential that this broader role is grounded in the core principles of medical physics and radiation science.

## Data Availability

N/A.
